# Navigating Access and Optimizing Medication Infusions in an Academic Medical Center: A Quality Improvement Study

**DOI:** 10.3390/pharmacy11040111

**Published:** 2023-06-30

**Authors:** Herolind Jusufi, Nicholas Boivin

**Affiliations:** 1 Department of Pharmacy, University of Wisconsin School of Pharmacy, Madison, WI 53706, USA; hjusufi@visanteinc.com; 2 Hackensack Meridian School of Medicine, Nutley, NJ 07110, USA

**Keywords:** pharmacy, specialty pharmacy, infusions, site of care, home infusion

## Abstract

(1) Background: The rising prices of medical infusions have resulted in the increased utilization of policies for payors to manage costs. These policies can be disruptive to the continuity of care, and health systems should develop a systematic strategy to address market changes and prevent patient leakage. (2) Methods: A quality improvement study was conducted by an interdisciplinary workstream to assess the current state of infusion services in an academic medical center in the Midwest and to provide recommendations for immediate access improvement and long-term system planning. An organizational assessment of the value stream was completed, which analyzed the available infusion capacity, billing strategy, patient mix/volumes, payor mix, staffing levels, and current policies. The interventions implemented after developing the infusion system strategy were triaging patients to the appropriate site of care to increase infusion capacity and eliminating paper orders in one of the health system’s Infusion Centers. (3) Results: Patients receiving medical infusions for oncologic conditions warrant unique considerations in evaluating the Infusion Center’s efficiency due to the infusion regimen’s length, complexity, and tolerability. The management of the payor site of care also poses a challenge for health systems to triage patients effectively without fragmenting care. (4) Conclusions: An organizational strategy around infusion services must include broad stakeholder representation to address the clinical, operational, and financial challenges to provide timely care to patients.

## 1. Introduction

The complexity of providing infusion services is challenging given the upfront considerations prior to the date of service. These factors involve the investigation of the benefits and prior authorization that need to take place for the patients. The investigation of benefits is the process where a health system will ensure that the desired outpatient services are contracted with the patients’ payor and any necessary pre-certification process is completed prior to the date of service to ensure that the patient is covered and the payor will reimburse the services. At Covered Entity A, the investigation of the benefits for drugs has been consolidated into the Medication Prior Authorization Coordinators (MPACs) within the pharmacy department, but it can still be difficult for patients to receive timely access to therapies given the administrative burden, including the need to complete a manual review process. As spending on medications has increased over time, with the average infusion costing between USD 32,000 and USD 136,000 yearly (depending on the medication and indication) [1,2,3,4], insurance companies have implemented various strategies to limit the costs, including prior authorization, step therapy, and formulary management [5]. More recently, additional measures including site of care restrictions [6] and medication bagging have been adopted as well.

Site of care restrictions limit the location where patients can receive an infusion that is clinically appropriate and in an alternative, lower-cost care setting. Patients will typically receive infusions in a hospital outpatient department (HOPD). An HOPD can provide closer monitoring of patients receiving infusions, and care can be escalated since they are often attached to a hospital. Additionally, an HOPD can charge a facility fee and an administration fee for a medication, which a non-HOPD cannot.

Other infusion service locations include professional/physician practices, standalone Infusion Centers, and home infusion. The total cost of an infusion between an HOPD and non-HOPD can differ by 80–150% based on the charge structure for medications and the inclusion of a facility fee [6]. Site of care restrictions limit patients’ access to care in health systems without non-HOPD facilities, which leads to patient leakage to obtain services elsewhere, and patients will often require an outside referral. Breaking the continuity of care in this way contributes to suboptimal outcomes for patients by diminishing close contact with their primary providers and to care not being documented centrally in the health system’s EHRs for all providers to see.

Medication bagging is an alternative to the traditional buy-and-bill model [7], where an Infusion Center purchases drugs themselves and bills the payor for the cost plus a markup. Medication bagging is less expensive to the payor since the drug is billed under the pharmacy benefit, rather than the medical benefit, which tends to have a lower charge structure. Different variations of bagging exist, which have implications for drug supply integrity and operational hurdles for health systems. White bagging is where a payor-mandated specialty pharmacy dispenses a medication for a patient and ships it to the site of care for administration [8]. Brown bagging is where a payor-mandated specialty pharmacy dispenses a medication for a patient and ships it directly to the patient, who then brings it into their site of care for administration. This is far less common as drug integrity is further reduced once a drug is dispensed to a patient. Clear bagging is where a health system’s own specialty pharmacy dispenses a medication and delivers it to the site of care.

Health systems prefer buy-and-bill from an operational and financial perspective. Drug integrity is ensured if a medication is shipped to the site of the Home Infusion Provider from the manufacturer/wholesaler. This cannot be guaranteed if a drug is shipped from an outside specialty pharmacy. Delays in therapy initiation while waiting on drug shipments and changes in therapy negatively impact care. It is also cumbersome to keep track of patient-specific inventory in the existing medication supply. Financially, dispensing of a medication outside of the health system reduces the margin of providing infusion services since there is a markup on a buy-and-bill drug, which accounts for the logistics, personnel time to compound/prepare the drug, and the necessary infrastructure in place to run a USP-compliant cleanroom. White/brown bagging removes this revenue stream from health systems, who are then only able to charge an administration fee for a drug. While clear bagging prevents delays in care by maintaining dispensing in-house, inventory is still segregated and tracked separately through patient-specific doses, which increases the operational burden to provide infusion services. Health systems must choose whether to negotiate with payors for a lower reimbursement rate to continue to buy-and-bill, accept alternatives such as white and clear bagging, or forgo treatment for these patients and refer them elsewhere. The American Society of Health System Pharmacists (ASHP) and the American Hospital Association (AHA) have written letters to the FDA to put a halt on white bagging as it poses patient safety risks and supply chain security risks since white bagging may not meet the provisions of the Drug Supply Chain Security Act (DSCSA) of tracking and tracing [9].

Free-standing or physician infusion clinics typically have a charge structure much lower than an HOPD at or near Medicare’s allowable rate (ASP + 6%) [10,11]. Moving to different sites of care also impacts the ability to maximize 340B savings depending on eligibility, which affects a health system’s ability to provide care to the widest reach of patients. These factors need to be taken into consideration for how facilities are classified and for strategies in the future as payors may implement site-neutral payment for infusion services.

Patient clinical factors have been considered in overall quality improvement efforts to address actionable factors that are a bottleneck to care. Compared to non-Oncology patients, Oncology patients tend to have a higher acuity of illness and longer infusion durations with multi-drug combinations and pre-medications and are more likely to experience adverse events during an infusion treatment [12], and infusions are tied to lab and doctors’ appointments. Because of these factors, Oncology patients have less flexibility than non-Oncology patients to navigate alternative sites of care, and preference is given to provide infusions within a hospital setting near their treating provider.

Drug pricing and the 340B program impact covered entities’ ability to “stretch scarce federal resources to reach more eligible patients and provide more comprehensive services”, including infusions [13]. Studies have shown that hospitals who participate in the 340B program are more likely to provide unreimbursed services, such as charity care [14,15]. One of the ways this is possible is because 340B drug discounts are typically 25–50% less expensive than what an organization would otherwise pay for a given drug [16]. Differing program statuses affect the ability to purchase drugs under a statutory price. Rural referral centers (RRC) and various other covered entity types are subject to orphan drug exclusions, meaning drugs with this FDA designation cannot be purchased at a 340B price [17]. Orphan drugs include those used for rare diseases, which encompass a large number used in Oncology care. Manufacturers may voluntarily elect to provide an orphan drug at a 340B-like price to a 340B-RRC-covered entity to retain market share; however, this pricing is not guaranteed, and agreements may be cancelled by manufacturers at any time. A disproportionate share of hospitals do not have orphan drug exclusion, but they are subject to group purchasing organization (GPO) prohibition, meaning that 340B-eligible outpatient departments cannot purchase medications from a GPO, as well as 340B [18]. Any covered entity, regardless of 340B status, must also decide whether to purchase 340B drugs for Medicaid patients. Statutes do not allow for duplicate discounts for a drug from both a covered entity and a Medicaid rebate, so organizations that elect to carve in Medicaid have an added layer of program complexity to ensure that reimbursement claims for Medicaid patients denote 340B purchases to prevent duplicate discounts.

Covered Entity A is an RRC, and Covered Entity B is a DSH. Covered Entity A elects to carve out Medicaid patients from their 340B program, while Covered Entity B is carved in. This posed complexity for the triage across all patient populations that was considered in the organizational assessment and recommendations for the future state of infusion services and patient triage. Infusion Centers that are non-hospital based are not eligible for 340B, including Infusion Center A and the Home Infusion Provider.

Another issue affecting access to care is the investigation of benefits process. Payors implement prior authorization, pre-certification, and pre-determination on high-cost infusion and clinic-administered meds, which can delay the initiation and continuation of patients’ therapies [19]. It is warranted to examine the workflows the MPAC team uses at Covered Entity A for improvements. Payors have improved processes for pharmacy benefits’ prior authorizations through electronic prior authorizations (ePAs), but this is limited to the medical benefit. Optimization in the investigation of benefits process was warranted to improve provider and prior auth team communication and serve patients in a timely manner.

The overall infusion space across the system of care was considered with expanding infusion drugs for both Oncology and non-Oncology patients. This included building additional infrastructure and partnering with existing providers to expand patient access. Home infusion was another strategy used to decrease the total cost of care and improve the patient experience by minimizing logistical considerations and providing infusion services directly at home. Covered Entity A is fortunate to already be partnered with the Home Infusion Provider, which has a robust program in place, as well as an ambulatory infusion suite on site (AIS). Continuous improvement of this relationship was paramount to provide additional capacity for non-hospital care to patients.

An interdisciplinary work group between the two Covered Entities’ Joint Operating Agreement (JOA) was formed to address patient access issues and to optimize infusion services across the enterprise. Participating members included nursing leadership, Covered Entity B leadership, Oncology leadership, pharmacy leadership, quality, safety, and innovation (QSI) leadership, and home infusion leadership. Other stakeholder groups engaged include authorization services, information technology (IT), the health system’s commercial health plan, and the Pharmacy & Therapeutics Committee (P&T).

The work group charter was organized into three phases: access phase, optimization phase, and long-term infusion services needs phase. The access phase sought to immediately improve patient access and minimize delays for infusion services. The optimization phase further exploited improvements from the access phase, as well as planned for an additional Infusion Center’s (Infusion Center F) design to meet patients’ needs. The long-term infusion services needs phase involved strategic planning for future workflows and monitoring tools to assess key performance metrics. Subgroups were formed to work on different pieces within their scope and will report to the larger work group.

Pharmacy leadership served as business partners to improve the medication use process, contracting, medication bagging, 340B considerations, the investigation of benefits, and other business issues as they arose. The role of the principal investigator was to act as a project manager under pharmacy leadership. This began with an assessment of the current state of infusion services compared to trends in the market. The principal investigator implemented immediate solutions and long-term strategies in collaboration with the work group.

The Health System has four Infusion Centers (A–D) within Covered Entity A. The Health System partners with a neighboring hospital (Covered Entity B) to extend infusion services through a Joint Operating Agreement (JOA). Infusion Center A is the sole physician-based Infusion Center in the system, while the rest are hospital-based. Covered Entity B operates a hospital-based Infusion Center, which also triages some patients to its Digestive Health Center (DHC) for infliximab infusions (regardless of indication). These two infusion locations are collectively referred to as Infusion Center E. The characteristics of all Infusion Centers are shown in Table 1 below.

## 2. Materials and Methods

An interdisciplinary workstream endorsed by senior leadership was created to assess the current state of infusion services and provide recommendations for access improvement and long-term system planning. The areas selected for evaluation and process improvement were based on group consensus by Infusion Centers and organizational leadership. This posed a risk of selection bias for the prioritization of efforts across patient populations based on the perceived need for concerted improvement efforts to expand infusion capacity. An assessment of the value stream was completed, which analyzed the available infusion capacity, billing strategy, patient mix/volumes, payor mix, staffing levels, and current policies. The data utilized included visit and billing information within the hospitals’ electronic health records, as well as pharmacy department wholesale catalogs to evaluate drug pricing and financial models across potential revenue and costs within different sites of care.

### 2.1. Current State Analysis

**Figure 1:**
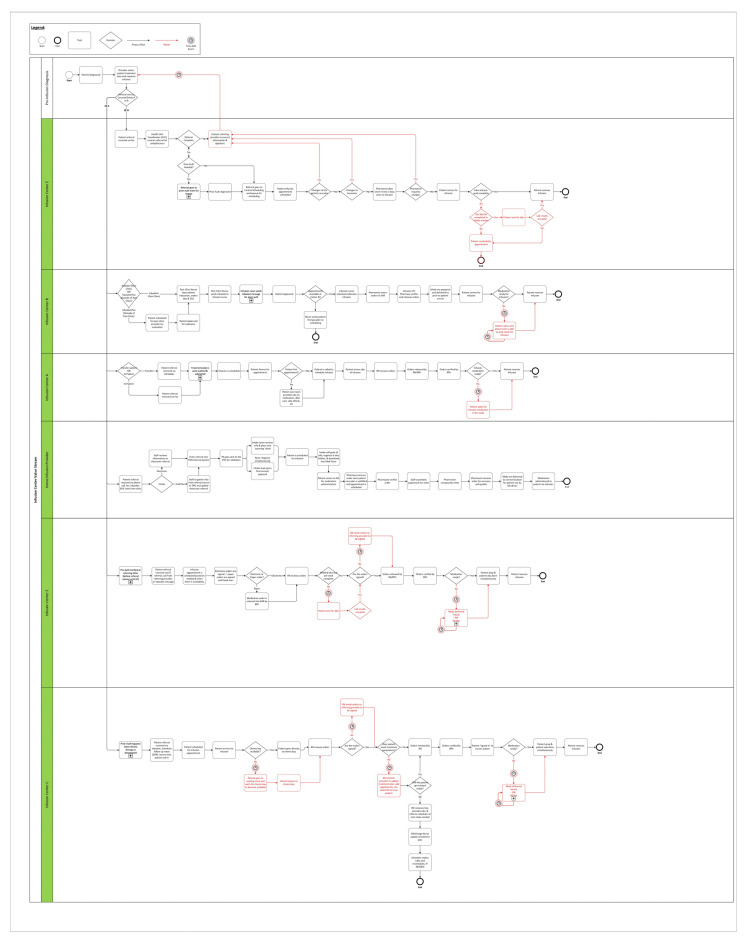
Infusion workflows and waste: in attached file.

**Figure 2:**
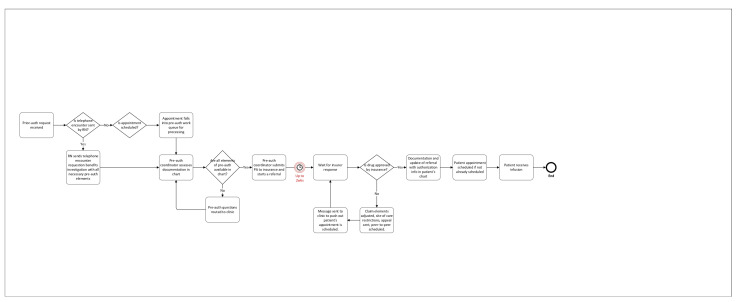
Pre-authorization workflow: in attached file.

A process map for the five Infusion Centers was created to identify bottlenecks from prescriber ordering of a drug through infusion of that drug (Figure 1). Workflows were further broken down into pharmacy workflows and prior authorization workflows (Figure 2). Process maps were created through the direct observation of work completed at each phase, as well as input from leaders and frontline staff.

Data were collected on the following areas of focus identified through the process mapping and strategic planning: capacity/hours of operation, encounter throughput, payer mix, drug sourcing, gross margin and 340B considerations, and patient scheduling. The data sources and HRSA orphan drug exclusion list are as follows.

Elements of the current state analysis included:

- Chair throughput;
- Anticipated patient volumes;
- Billing classification;
- Payer mix and alternative sites of care;
- 340B cost savings;
- Home infusion provider ambulatory infusion suite impact.

### 2.2. Site of Care Analysis

Orders and charges data for both Covered Entities were generated from the electronic health records. Infusion Center A’s data were pulled separately from other Infusion Centers since their charge structure is physician-based as opposed to hospital-based for the remainder.

The optimal site of care was determined based on the proximity of providers, Infusion Center capabilities, level loading of patients across Infusion Centers, and 340B cost savings.

ASP-based reimbursement was modeled across all sites to determine medication shifting opportunities. Data from the 2019 Advisory Board Market Scenario Planner tools were used to project infusion growth at 5 and 10 years from 2021 [20]. This considered location, age, sex, payor, and disease state. Data from the 2018 Advisory Board Infusion Center Pro Forma were used to estimate reimbursement rates relative to ASP for commercial and governmental payors [21]. From here, the patient mix by each site was determined using the Routed Charges Report. ASP-based reimbursement was determined using the same report based on the quantity of billing units billed for each drug and drug cost subtracted based on 340B classification. Table 2 below shows 340b characteristics for each infusion center, Table 3 summarizes ASP multipliers based on payer type and type of reimbursement. This information is then used for Table 4 which shows the difference in reimbursement rates by site of care and 340b utilization. This determined the estimated margin for each Infusion Center. While Covered Entity A’s Infusion Centers are Medicaid carved out, the dataset modeled carved in status. Table 5 shown below shows estimated growth for chemotherapy and non-chemotherapy patient populations with Table 6 showing growth by infusion center.

Medications were grouped into categories based on the medication infused and provider specialty:

Provider groups:

- Hematology/Oncology;
- Pain;
- Nephrology;
- Gastroenterology;
- Infectious Disease;
- Internal Medicine;
- Neurology;
- Pulmonology;
- Rheumatology;
- Family Medicine;
- Allergy;
- Endocrinology;
- Dermatology;
- Other.

The following were used as measures of Infusion Center throughput:

1. Appointments per day—the number of patients served in an Infusion Center on a given day. Data were stratified by weekday and weekend hours for Infusion Centers open on the weekends.
2. Appointments per chair per day—the number of appointments divided by the number of chairs in an Infusion Center.
3. Appointments per RN FTE per day—the number of appointments divided by the average number of RN FTEs.

Data from the 2018 Advisory Board Infusion Center Pro Forma were used to estimate the reimbursement rates relative to ASP for commercial and governmental payors [21].

Only drug reimbursement was considered for the analysis, and CPT reimbursement was excluded since the payors are moving to make the reimbursement site of care neutral.

Data from the 2019 Advisory Board Market Scenario Planner tools were used to project infusion growth at Years 5 and 10 from the baseline. Infusion Centers A and C use Oncology growth rates, and all other sites use non-Oncology growth rates in the analysis. The projected Infusion Center growth was then estimated on top of the organic growth rates based on the anticipated service expansion for different provider groups.

This would determine future capacity needs over a 10-year period against planned organization growth for HOPD and non-HOPD chairs.

### 2.3. Alternative Sites of Care Workflow

Referral workflows for an ambulatory infusion suite (AIS) were created through a subgroup of the infusion work group. The subgroup identified patients to refer and operationalized those changes to meet stakeholder needs. Key considerations for referrals included payer considerations, medication selection, and referral workflow.

## 3. Results

### 3.1. Current State Analysis

The throughput measures are as follows. Table 7 summarizes the number of chairs by infusion center and day of the week, Table 8 summarizes the hours each infusion center is open, and Table 9 shows how many appointments each infusion center has comparing weekdays to weekends. Table 10 and Table 11 takes information from Table 9 to find the average number of appointments per day in each infusion center and the number of appointments per day on average for each chair, respectively.

The chairs available helped predict the chair needs in the future with patient growth.

The hours open daily shows that most Infusion Centers are open for 9 h days, while Infusion Centers D and E have extended weekday hours.

Annualized appointments are greater for those Infusion Centers with more chairs (Infusion Centers C and E).

The appointments per day were stratified by weekdays and weekends since staffing levels and patient volumes differ, which can skew the results if they are rolled up.

The appointments per chair per day is a throughput measure showing how often a chair within an Infusion Center is cycled. Infusion Centers that are open longer (Infusion Center D and Infusion Center E) and whose average infusion time is shorter (Infusion Center B) tend to have a higher measure.

Table 12 shows the average RN FTEs per day which were provided by nurse managers for the respective Infusion Centers. Larger Infusion Centers (Infu-sion Centers B and C) and those serving Oncology patients (Infusion Centers A and C) tend to have higher staffing needs.

Table 13 shows the appointments per RN FTE per day. The table shows the number of appointments any given RN is responsible for in an 8 h shift. These were stratified by weekdays and weekends as staffing and patient volumes differ.

### 3.2. Growth Measures

Table 14 summarizes the projected growth in appointments for each infusion center. Infusion Center A shows the largest growth, which is driven by payor preference for alternative sites of care. These data helped predict future chair needs based on current chairs within the system. Both Covered Entities A and B prohibit brown and white bagging due to concerns with supply chain integrity. A subset of patients receives clear-bagged medications from the health system’s own specialty pharmacy. Managed care contracts with various commercial payors have different stipulations that state payors will not conflict with Covered Entity A’s policies. Consideration was given to site of care redirection for non-hospital-based infusion chairs. Specialty pharmacy requirements were not an emphasis of the project.

### 3.3. Site of Care Analysis

Table 15 summarizes 340B classification by provider group types. Hematology/Oncology/BMT, Gastroenterology, Neurology, and Rheumatology all have high-cost medications that are orphan drugs. Based on the data gathered, it would be most advantageous to provide infusion services for these patients in a 340B DSH HOPD. Other non-Oncology specialties such as Pulmonology, Dermatology, Allergy, and Nephrology do not pose as many orphan drug exclusions as do the previous specialties mentioned, which warrants infusion at either a 340B RRC HOPD or a 340B DSH HOPD. Infectious Disease and Pain patients utilize low-cost drugs in comparison to specialty patients, which did not show as large of a fiscal impact if infused in an HOPD or non-HOPD, as well as drug cost savings with 340B in the analysis. 

### 3.4. Chair Analysis

Table 16 below is summarizes payer mix by infusion center. Table 17 shows the estimated amount of project chairs needed at baseline, year 5, and year 10 based on number of expected appointments.

The projected chairs needed measure grows proportional to the projected appointment growth over the 10-year period.

The projected chair type needed builds off of Table 18 to show the predicted HOPD and non-HOPD chair mix required to continue to serve patients within the Covered Entities’ JOA. These were taken into consideration with planned growth to ensure capacity could be maintained without losing non-HOPD patients.

Table 19 shows the projected chairs needed by provider group measure breaks down overall chair needs across specialties, which was considered for level loading of patients across the system in the future state. Hematology/Oncology requires >50% capacity within the system, with Pain, Infectious Disease, Rheumatology, Gastroenterology, and Neurology following. Infusion Centers with mixed patient populations (Infusion Centers A, D, and E) treated every infusion equally to roll up into the number of chairs required for the analysis. This was a limitation since infusion times differ for medications, especially when comparing Oncology to non-Oncology.

Table 20 summarizes the original planning had Infusion Center F open as an entirely HOPD Infusion Center with the sunset of infusion services at Infusion Center A. The Home Infusion Provider is included in future chair planning as they have become more integrated in the infusion system services because of the project. Overall chairs in the system increase, but non-HOPD chairs decrease because of Infusion Center A’s closure. There was uncertainty as to how many chairs would remain open at Infusion Center C with Infusion Center F’s opening. A dual-hub model for infusion services was planned, but there may be a change in facilities maintained at UH, which is why a range of chairs from 0–41 is shown.

Table 21 is based on recommendations to split Infusion Center F into an HOPD/non-HOPD Infusion Center were provided by the pharmacy business leads. This shows increased overall infusion capacity across the system while growing non-HOPD chairs to align with projected needs by 2031 of at least 21 chairs.

The projected capacity of new infusion centers used a weighted average of Infusion Center C’s and Infusion Center A’s throughput to project this for Infusion Center F is summarized in Table 22. A target of three appointments per chair per day was predicted for infusion capacity at the Home Infusion Provider based on the non-Oncology patient population served.

### 3.5. Alternative Sites of Care Workflow

The Home Infusion Provider payor considerations are:

- Medicare/Medicare Advantage—regulatory limitation due to no provider on site;
- Medicaid/Medicaid Managed Care—regulatory limitation due to no provider on site;
- Commercial—variable.

#### 3.5.1. Ocrelizumab Referral Workflow (Figure 3)

Referrals were generated based on patients receiving non-Oncology infusions at Infusion Center A. Appointments for ocrelizumab were extracted to determine the Home Infusion Provider’s insurance eligibility prior to sending referrals for the investigation of benefits. Additionally, the prior authorization team identified patients at the time of the investigation of benefits for new and existing patients to be referred directly to the Home Infusion Provider.

#### 3.5.2. Specialty Referral Workflow

The approach for ocrelizumab was expanded to include all specialty, non-Oncology drugs, referred to as “Outside Infusions (OSI)” at Infusion Center A. Paper order sets were updated as needed to meet the needs of the Home Infusion Provider and regulatory requirements. The principal investigator contacted new clinics that had not previously received referrals from the Home Infusion Provider and created a buy-in for site of care changes. It was the clinics’ responsibility to contact the patients for a warm handoff that their infusion site would be changing, as well as faxing paper orders to the Home Infusion Provider.

Medications included:

- Immune globulin (IVIG);
- Infliximab and biosimilars;
- Vedolizumab;
- Abatacept;
- Belatacept.

#### 3.5.3. Lidocaine Referral Workflow

Referrals were initiated by Infusion Center B’s staff for established patients on maintenance therapy at Infusion Center B. Lidocaine paper order sets were updated to meet the Home Infusion Provider’s and regulatory needs. The Home Infusion Provider worked directly with the Pain Management Clinic to obtain faxed orders and handle patient handoffs.

A 6-month ramp up to a goal of 100 infusions monthly was estimated based on infusion capacity at the Home Infusion Provider. A patient on maintenance therapy for lidocaine can receive infusions weekly, biweekly, or monthly. It was assumed that a patient would receive infusions biweekly for the ramp up and that 3 patients would be referred to the Home Infusion Provider in Month 1, and 5 additional patients would be referred monthly thereafter until they reached capacity. An average of three infusions per patient monthly was assumed based on the current average frequencies at Infusion Center B. Table 23 shown below shows the estimated number of patients and infusions by month based on the ramp up.

## 4. Discussion

### 4.1. Current State Analysis

Chairs, hours of operation, and weekend availability all differed between Infusion Centers. Infusion Centers A-C are open M-F for 9–10 h days. Infusion Centers D and E are open for 12+ hour days on weekdays and have weekend availability also. Infusion Center size correlated with the number of appointments also with Infusion Centers C and E having the highest weekday volumes (65.9 and 67, respectively). A more representative measure of efficiency was appointments per chair per day. This measured how many appointments on average took place for each available chair/bed in an Infusion Center. This considered how long an Infusion Center was open, any delays in appointments, and appointment duration. Oncology presented higher-acuity patients with increased pre-medication, the number of infused drugs, and the recovery time post infusion than their non-Oncology counterparts. This was reflected by a lower throughput of 2.1 and 1.6 appointments per chair per day for Infusion Centers A and C, respectively. Infusion Center A is a physician-based Infusion Center, which takes lower-acuity patients than Infusion Center C, which is likely why their throughput measure is higher. In addition to this, 4–5 of the 17 chairs at Infusion Center A are occupied by outside infusions daily for non-Oncology patients who have site of care restrictions that require infusions in a non-hospital-based site of care. Advisory Board benchmarks showed 2–3 infusions per chair per day as the 50–75% percentile for non-Oncology patients and 1.5–2 for Oncology patients [20]. Smaller Infusion Centers benefit from an increased turnover of chairs due to the ease of managing a smaller operation. Larger Infusion Centers such as Infusion Center C experience diseconomies of scale with 41 chairs/beds that require increased coordination and patient/provider triage to turnover chairs in a timely manner. Infusion Centers D and E have longer hours of operations, and their appointments per chair per day are also higher. This suggests that additional scheduling availability through a longer appointment day provides less “crunch time” between peak times around 10 AM–2 PM. This allows for more patient flexibility to schedule in the evening and improve overall Infusion Center throughput. The validation of these hypothesis can be performed through the collection of other throughput measures including pharmacy order delays, patient wait times, and delays in patient arrivals. These are future datasets that can be estimated for process improvement in Infusion Center efficiency and patient satisfaction to see if a 12 h infusion day schedule is optimal to spread out patient appointments and fill more appointments in each chair daily. Appointments per chair per day is not representative of Infusion Center efficiency for Infusion Centers D-E on weekends (both 1.1), since they do not actively use all available chairs. This can be seen with 1.5 and 2 RN FTEs on weekends, respectively. This suggests that patients do not desire weekend infusions since there is excess capacity at these sites on Sat and Sun, while Infusion Centers experience heavy delays during desirable hours M-F. This warrants additional investigation of the appointment offerings and providing incentives for patients to schedule away from 10 AM–2 PM on weekdays or move to weekend infusions when possible. Based on expert opinion, the work group did not predict that providing additional weekend hours to other Infusion Centers would have a large impact on patient satisfaction as a short-term solution to increasing Infusion Center capacity.

Registered nurse (RN) daily appointments were another measure to gauge staffing adequacy where efficiency is concerned. Advisory Board benchmarks place 4–6 appointments per RN per day in the 50–75% percentile of efficiency [20]. These values are reflected in the current Infusion Centers’ numbers. Infusion Center C lags other centers for the number of appointments per RN FTE daily, and this can be attributed to longer patient appointment times and higher acuity, which warrant a lower patient: nurse ratio. A given infusion nurse has the capacity to care for 2–3 patients simultaneously. Most of the work by nursing is performed at appointment initiation and ending; however, higher-acuity patients require further monitoring and triage for infusion tolerance and reactions also.

Infusion appointment growth is projected to be higher than organic growth outside of HOPD Oncology due to planning for additional Oncology and non-Oncology clinic services, which corresponds to an increased number of patients and requirements for infusion therapy. Infusion Center A leads anticipated growth efforts with 21.8% growth and Infusion Center C the least with 5.5% growth. This is partly due to the site of care shifting of patients away from hospital-based infusions, which affects Infusion Center growth planning to account for additional non-HOPD chairs in addition to more chairs overall.

Drug sourcing and site of care redirection are two payor strategies to lower cost to health plans by shifting revenue away from a health system through the pharmacy benefit and a lower cost structure, respectively. The former presents challenges in drug supply chain integrity, which is why Covered Entities A and B prohibit white and brown bagging. Covered Entity A has been shielded from commercial restrictions through its own internal commercial plans and is now seeing the impact from other national commercial payors, who are increasingly adding drugs to these alternative lists. An additional investigation to quantify the impacted patients and those that were forced to be treated out-of-network is imperative to show the financial impact the Covered Entities are having based on these payor practices.

Order workflow and the prevalence of paper comprised another area of opportunity in optimizing infusion services between Covered Entity A and Covered Entity B. In the present state, all orders sent to Covered Entity B are received through a paper fax, which is transcribed into Covered Entity B’s electronic health record, which are separate instances from each other. Considering Covered Entity B is the largest Infusion Center in terms of volume, this is a great source of error and waste within infusions as problems can occur in order entry, transmission, transcription by Covered Entity B’s infusion pharmacist, etc. Electronic health record companies have built infrastructure for therapy plans, which are order sets that can be used across multiple encounters and have medication and non-medication orders built into them for infusion services outside of Oncology. Covered Entity A does not have robust therapy plans built into its systems currently, but Covered Entity B’s Epic does. Therapy plans cannot be transmitted across different Epic instances, so there are multiple options for the future state of workflows. The first is that therapy plans are built into Covered Entity A, and the ordering provider enters these orders electronically. These orders can then be printed on paper and faxed to Covered Entity B for transcription into Covered Entity B’s system. The advantage of this is increased controls on ordering through electronic means, and there is not a need for a provider to interface between two separate electronic health record instances to care for patients. Therapy plans will eventually be needed in Covered Entity A’s Link anyways if additional non-Oncology infusions revert to Covered Entity A from Covered Entity B with the opening of Infusion Center F, so building them sooner rather than later will allow for the seamless transitioning of patients. The downside of this approach is the maintenance of faxed paper orders and errors that can still occur upon transcription between Covered Entities. The alternative approach is to require providers to enter electronic orders in Covered Entity B directly. This removes any manual transcription/interpretation of orders and allows for electronic controls of ordering that are superior to paper. The downsides of this are provider desirability to enter multiple EHRs to enter orders to learn two nuanced systems. Clinic RNs enter in orders for providers on their behalf, and the provider verifies/signs the orders. To facilitate the same process in Covered Entity B, both providers and clinic RNs would need access to Covered Entity B’s EHR, which may prove an IS risk to provision access to hundreds of additional people.

Prior authorization services for medications are provided by Medication Prior Authorization Coordinators (MPACs), which is internal to the pharmacy department. The infusion workgroup investigated how these workflows could be improved to decrease wait times to therapy initiation (Figure 2). The largest bottleneck to initiating infusion identified were waiting for insurer response, which takes up to 2 weeks at times. The literature has shown that prior authorizations delay patient care, can lead to higher glucocorticoid exposure in patients awaiting an infusion for a rheumatologic condition, and lead to wasted physician time [23,24,25,26,27,28,29]. Prior authorizations for the medical benefit are still largely manual and not automated and electronic as pharmacy benefit authorizations have been. This is due to many documentation requirements that must be submitted and evaluated prior to patients starting high-cost therapies. MPACs and clinics have close coordination for clinics to submit information to MPACs to complete a timely investigation of benefits as soon as it is known that a patient requires infusion therapies. MPACs are also notified when a patient on a high-cost drug is scheduled for infusion so that they can ensure the investigation of benefits is complete if the clinic did not already send advanced notification. Infusion Center pharmacists are another stop gap in the process to verify orders for infusion medications and clinically administered medications, which provides another necessary stop gap to ensure the authorization for the drug is in place to prevent negative financial impact to the health system and patient. Based on the current level of service, no immediate process changes were noted for pre-authorization workflows.

### 4.2. Site of Care Analysis

#### Chair Analysis

**Figure 4:**
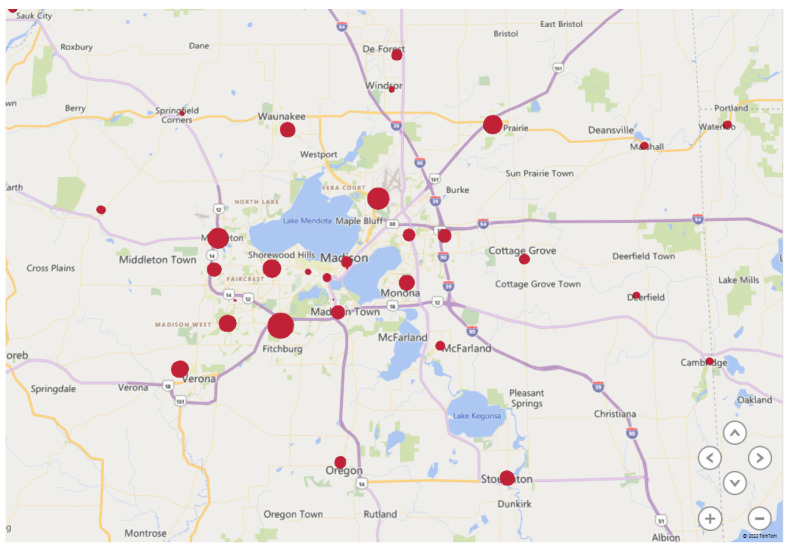
Dane County area Oncology zip code analysis.

**Figure 5:**
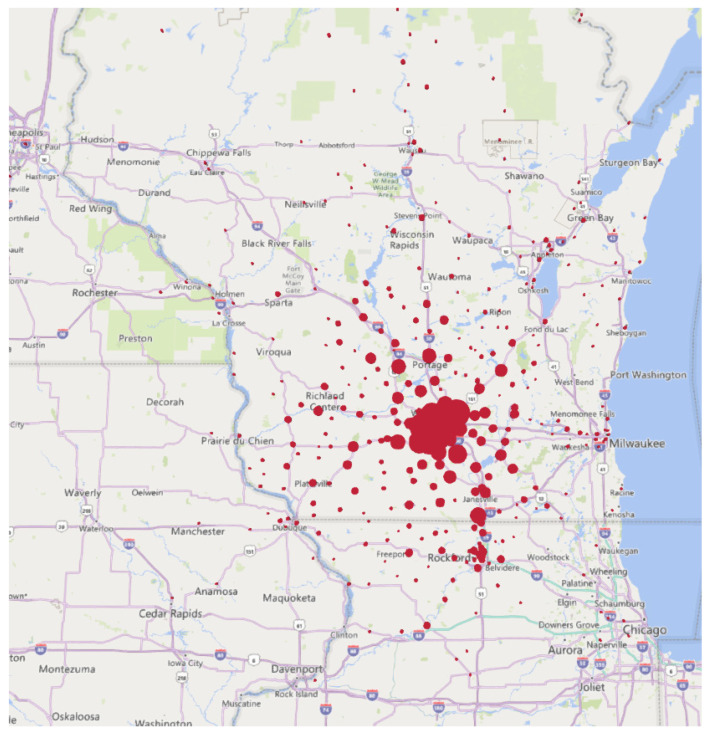
Wisconsin–Illinois Oncology zip code analysis.

Assuming the current Infusion Centers cannot take on additional appointments with existing infrastructure, there is a need to expand by at least 13 chairs within the next 10 years (94 to 107). Within this model, HOPD and non-HOPD chairs must be considered due to increased payor preference for alternative sites of care outside of the hospital-based setting. The estimated split was at least 86 and 21 chairs, respectively, based on anticipated Infusion Center growth. The opening of Infusion Center F will provide 72 chairs in the system at the expense of Infusion Center A’s closure and the consolidation of chairs at Infusion Center C. Future chair capacity at Infusion Center C is still indeterminate, but estimated to continue to serve at least 25–50% of Oncology volumes in the future state as a dual-hub model for Oncology services. Figure 4 and Figure 5 shows a zip code analysis for Oncology patients in the Dane County area and all of WI and IL. Those in the Dane County area are about 50% split between the east and west side of Madison for proximity to the dual-hub model. Having most Oncology services at Infusion Center F is advantageous for those who travel for care from northern Illinois and surrounding areas in WI as it is located near a major interstate. Oncology has an estimated need for 65.6 chairs between HOPD/non-HOPD by Year 10, so 16–32 chairs would need to remain at Infusion Center C to continue to provide services to 25–50% of the total Oncology patients at Covered Entity A.

The original planning for Infusion Center F included all HOPD chairs. Many patients would have been lost to the system with this plan due to Infusion Center A’s closure and the lack of alternative sites of care within the system outside of six chairs at the Home Infusion Provider. The Home Infusion Provider does not have the skill mix to take Oncology patients either, so any Oncology patient with site of care restrictions would be treated outside of Covered Entity A.

The updated Infusion Center F planning split the Infusion Centers into a 50/22 split with HOPD and non-HOPD chairs. This ensures adequate chair types to serve Oncology and non-Oncology site-of-care-restricted patients across the system that the Home Infusion Provider alone cannot. The minimum amount of HOPD and non-HOPD chairs needed by Year 10 is estimated to be 86 and 21, respectively. The updated Infusion Center F planning provides for at least 86 HOPD chairs (more depending on how many chairs remain at Infusion Center C) and 28 non-HOPD chairs. Infusion Center F will also be built in such a way that additional chairs can flip to non-HOPD over time to allow for maintenance of patients without leakage from the system.

The projected capacity of infusions at Infusion Center F was taken through linear extraction of HOPD chairs at Infusion Center C and non-HOPD chairs at Infusion Center A. This yielded an overall estimated appointments per chair per day of 1.7, with overall appointments at 121.3 daily between HOPD and non-HOPD Infusion Centers collectively. This was based on weekday hours and a 9–10 h Infusion Center day. Similarly, the Home Infusion Provider is estimated to be able to take on three appointments per chair per day as a benchmark for non-Oncology infusions, and the coincides with the current throughput of non-Oncology Infusion Centers. These data will allow for volume planning over time when Infusion Center F opens and current planning for patient shifts from Infusion Center A and Infusion Center B to the Home Infusion Provider.

Dual-Infusion Centers located in the same vicinity at Infusion Center F have special consideration to be compliant with regulations. This includes separate entrances, no patient/staff mixing, and separate staffing between Infusion Centers. Pharmacy operations are still able to be provided through a single cleanroom, which will be built in Infusion Center F.

### 4.3. Alternative Sites of Care Workflow

The capabilities of the Home Infusion Provider were determined prior to initiating referrals for infusions. Their ambulatory infusion suite has six chairs, which are open weekday hours, like Infusion Center A. Skill mix includes nursing with experience in specialty infusions, but not chemotherapy. The Home Infusion Provider does not have a provider on site overseeing infusions, so they are carved out from Medicare/Medicaid due to a regulatory limitation that requires a provider on site. While estimated infusion capabilities are ~18 infusion daily based on six chairs, they currently operate with one RN FTE daily due to low volumes. Careful consideration in ramp ups for a single nurse to adequately provide infusion was given. The future state for the Home Infusion Provider will be to incorporate two FTE daily like Infusion Center B to maximize appointments in their available chairs. The future state includes a provider on site as well so that additional governmental patients can be referred also.

The incorporation of the Home Infusion Provider into the infusion services is primarily for ambulatory infusion suite patients. While the Home Infusion Provider provides home infusion services, it was not pursued as a part of the project due to nursing agency preference to provide short, frequent infusions (i.e., chronic antibiotics and TPNs). Certain specialty infusions have been demonstrated to be safe and effective in the home setting, but these take longer to infuse (1–4 h) and are shorter in frequency (every 4–8+ weeks). The future direction could investigate moving patients from provider-infused medications to those that can be self-administered and on the pharmacy benefit such as subcutaneous immunoglobulin.

Referrals to the ambulatory infusion suite at the Home Infusion Provider came primarily from outside infusion patients at Infusion Center A. These patients present a capacity constraint to Infusion Center A due to their growing Oncology population. Infusion Center A is the only non-HOPD within the system, so expanding additional alternative sites of care allows for increased patient convenience to be able to schedule appointments and prevent leakage from the system. Current outside infusions seen at Infusion Center A include IVIG, ocrelizumab, infliximab (and biosimilars), belatacept, abatacept, and vedolizumab. IVIG and ocrelizumab present the highest yield per infusion at Infusion Center A due to their prolonged infusion times compared to other drugs, so these were selected for initial referrals to the Home Infusion Provider. This would free up the infusion schedule at Infusion Center A the most compared to other infusions for the time it takes to coordinate patient shifting between sites. The Home Infusion Provider has its own ambulatory contracts with commercial payors, which were taken into consideration for referrals as well.

Infusion referrals started with ocrelizumab as a small test of change due to its infrequent administration for maintenance doses (every 6 months) [30]. This would allow for testing of the workflow and making modifications as necessary while maintaining patient satisfaction.

### 4.4. Specialty Referrals Workflow

The principal investigator identified patients already scheduled at Infusion Center A to refer to the Home Infusion Provider for the investigation of benefits. If referrals are accepted, the Home Infusion Provider contacts the patient’s clinic to fax paper orders over and to contact the patient that his/her site of care would be changing from Infusion Center A to the Home Infusion Provider for their next infusion. The Home Infusion Provider would then contact the patient to schedule an appointment and answer any questions. The Home Infusion Provider proceeds to provide the infusion service to the patient and fax a paper documentation form back to the patient’s clinic with each infusion noting what was infused and any pertinent notes such as infusion reactions.

Patient buy-in occurred best when the clinic contacted the patient directly to explain the need for a site of care change. This also provided a warm handoff to the Home Infusion Provider to ensure smooth transitioning of patients between sites. Paper orders could not be avoided with the Home Infusion Provider since they are not in Covered Entity A’s EHRs. This is primarily due to the EMRs not having a robust ERP system for home infusion to incorporate clinical documentation, charging, and drug dispensing workflows in a singular system. The Home Infusion Provider thus uses a system in the referral workflow.

As an ambulatory infusion suite, the Home Infusion Provider cannot bill for oral pre-medications including acetaminophen and diphenhydramine. Patients were initially required to supply their own from home, but this was a dissatisfier to the overall patient experience as many did not keep these medications in house. An adjustment to the workflow that was made was the Home Infusion Provider providing oral pre-medications free of charge to patients given that the cost is low and the perceived impact to the patient experience is high.

Once referrals were optimized after 6 months with ocrelizumab and IVIG, referrals for any non-Oncology medication from Infusion Center A began. Covered Entity A’s internal prior authorization team (MPAC) also referred patients directly to the Home Infusion Provider for new patients who were identified as having site of care restrictions, which eliminated a patient needing to be seen at Infusion Center A entirely. This also presented another benefit to patients by having less changes in sites of care. Loading doses/initial infusions for drugs remained in an HOPD setting (Covered Entity B), and subsequent maintenance doses can be shifted to the Home Infusion Provider instead of Infusion Center A when payors require an alternative site of care.

### 4.5. Lidocaine Referrals Workflow

Patient wait times to initiate and schedule for lidocaine infusions at Infusion Centers B and D were estimated to be between 3–6 months based on the backlog of patients. This presents an immediate access issue to the system to provide treatments to chronic pain patients who otherwise rely on excessive doses of opioids, which have negative side effects. Based on the site of care analysis, lidocaine infusions can be provided irrespective of site of care due to their low ingredient cost and CPT reimbursement approaching site neutrality.

Lidocaine referrals were made like specialty medication referrals. However, it was determined by the infusion work group that only established patients would be referred to the Home Infusion Provider, and no new starts would occur there. Patients have the highest chance of not tolerating therapies at initiation, and an EKG is performed at baseline. These are best managed with a provider on site at Infusion Center B. The Home Infusion Provider will serve as a hub for established patients to receive maintenance infusions and have higher flexibility in schedule since Infusion Centers B and D are at capacity and cannot accommodate the many patients waiting to start therapy.

An unanticipated barrier to lidocaine referrals was drug shortages, with the lidocaine preservative-free 2% product and alternatives used to make lidocaine infusions going on shortage at the onset of the Home Infusion Provider referrals. This will be taken into consideration to ramp up infusions at the Home Infusion Provider at a sustainable rate, which can provide infusions to patients without exhausting the available drug supply on allocation.

### 4.6. Future Directions

#### 4.6.1. Scheduling and Chair Time

The analysis of the current state workflows identified different practices in patient scheduling. Different strategies for scheduling patients included setting parameters on which types of infusion can be scheduled during specific intervals in a day, scheduling by appointment length, scheduling by provider, and many others. Infusion Center E utilizes central scheduling to put patients on the schedule, whereas Infusion Centers A–D use internal scheduling teams. Infusion Center C uses a first-come, first-serve model up to a maximum number of appointments per day and then uses manager approval for additional appointments to be added. Each appointment type is associated with a certain appointment length, and a summation of appointment lengths day to day is tracked to plan for nursing staff needed to accommodate all patients. Scheduling at Infusion Center B is most straightforward since they only infuse lidocaine, and each appointment is scheduled a standard 2 h. This coincides with the highest throughput of all Infusion Centers due to low variability in drug and appointment length.

Scheduling software exists to attempt to take all resources (chairs, nursing FTEs, pharmacy capabilities, appointment length) and use algorithms to identify an optimal schedule that maximizes the number of appointments and decreases patient wait times. These software packages were actively being investigated by several Infusion Centers within the system for future implementation. A challenge presented with scheduling that is unique to Oncology patients is linked appointments for lab–MD–infusion. Schedulers must contend with additional resource availability to navigate a patient between three locations on a given day without excessive waits between appointments. MD clinics generally prefer to schedule patients for infusion in the morning, which creates a bottleneck around peak times of the day between 10 AM and 2 PM, where Oncology patients flood the Infusion Center at a greater rate than can be accommodated by both nursing and pharmacy resources. This leads to excessive patient wait times, which last through the rest of the day. Strategies to streamline when MD appointments occur, unlinking lab–MD to infusion appointments, are additional ways to be pursued to optimize infusion scheduling for Oncology patients.

#### 4.6.2. Therapy Plans for Non-Oncology Infusions

As discussed previously, therapy plans for non-Oncology infusions will improve care coordination and decrease errors. IT is actively being engaged to recruit resources to build therapy plans in Covered Entity A’s EHRs for medical patients, as many will move to Infusion Center F from Covered Entity B.

#### 4.6.3. USP 797/800 Operations

**Figure 6:**
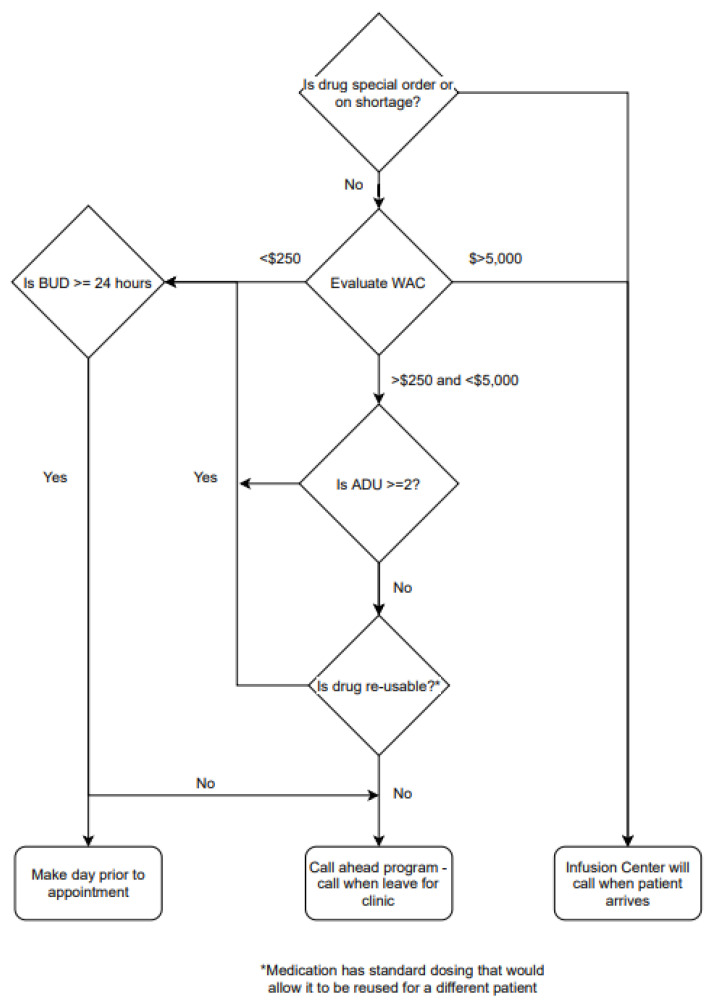
Drug pre-mixing flowchart.

Pharmacy operations play an integral role in infusion services’ planning and providing safe and effective medications to patients without excessive delay. Pre-mixing of medications decreases wait times at the expense of increased waste for no-shows, cancelled appointments, and therapy changes. The criteria for the incorporation of new medications into existing pre-mixing were created (Figure 6). Further work to optimize medication preparations and deliveries should be considered with additional infusion services’ planning for Infusion Center F.

#### 4.6.4. Assessment of Therapy Continuation

The principal investigator sought to figure out how to provide infusion services. It is also warranted to investigate why we should provide infusion services. Infusions such as lidocaine are provided to patients indefinitely without set criteria for therapy continuation, and patients may be unnecessarily receiving care, which represents waste in the system. Additionally, certain medications have take-home alternatives that can be prescribed on the pharmacy benefit and administered by the patient outside of an Infusion Center, which opens capacity for patients who require services. Expanding efforts to de-prescribe is another strategy that should be pursued moving forward. De-prescribing has been found to improve health-related quality of life and decrease costs in older adults [31]. It is possible that this outcome would hold true outside the older adult population as well.

### 4.7. Limitations

The health system process improvement efforts in the evaluation of the current state and implementation of initiatives to expand infusion capacity are unique based on established processes and the patient population served. As a result, this study may not be exhaustive of all areas that health systems should address when evaluating infusion strategy, and others may need to prioritize efforts based on short- and long-term needs to address challenges to patient access to care.

The impact on out-of-pocket expenses for patients moving from a higher to a lower site of care was not evaluated as a part of this study. This can certainly impact the affordability of care, and patients may prefer to proactively be treated at the most-cost-effective site of care when presented with multiple options.

## Figures and Tables

**Figure 3:**
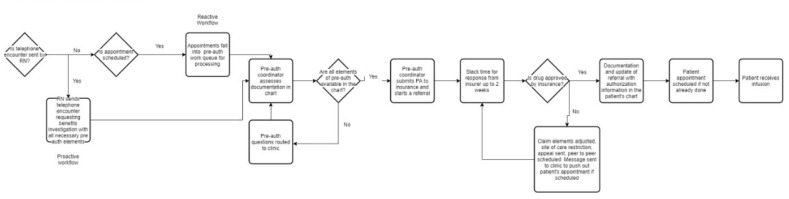
Ocrelizumab referral workflow: in attached file.

**Table 1:** Infusion Centers’ Characteristics.

Infusion Centers’ Characteristics			
Characteristics	Infusion Center A	Infusion Center B	
Patient Populations	1. Heme/Onc/BMT 2. Outside Providers	1. Pain (lidocaine)	
Billing Classification	Physician-based	Hospital-based	
340B Status	Not eligible	RRC	
Number of Chairs	17	5	
Hours	M-F 0800-1700	M-F 0800-1700	
**Infusion Centers’ Characteristics**			
**Characteristics**	**Infusion Center C**	**Infusion Center D**	**Infusion Center E**
Patient Populations	1. Heme/Onc/BMT	1. Heme/Onc/BMT 2. Pain (lidocaine) 3. UH Clinics	1. Infectious Disease 2. Specialty Providers
Billing Classification	Hospital-based	Hospital-based	Hospital-based
340B Status	RRC	RRC	DSH
Number of Chairs	41	8	18 + 5 ^1^
Hours	M-Th 0800-1800 F 0800-1700	M-F 0800-2000 Weekend/Holiday 0800-1400	M-F 0730-1930 Weekend/Holiday 0700-1530

^1^ Five additional chairs within a nearby digestive health center.

**Table 2:** Infusion Centers’ 340B Characteristics.

	Infusion Center A	Infusion Centers B, C, and D	Infusion Center E
340B Classification	Not eligible	^1^ RRC	^2^ DSH
Exclusions	N/A	Medicaid Carved Out	None
		Orphan Designated Drugs	

^1^ Rural Referral Center; ^2^ Disproportionate Share Hospital.

**Table 3:** Payer ASP Multiplier Table ^1^.

Payer	HOPD Reimbursement ^2^	Freestanding Reimbursement
Commercial	1.5	1.65
Medicaid	0.85	0.85
Medicare	1	1
Health System Commercial Plan	1.5	1.65
Self-Pay	1.1	1.1
Other	1	1

^1^ Average Selling Price; ^2^ Hospital Outpatient Department.

**Table 4:** Medicare Reimbursement by Site of Care and 340B Utilization.

	HOPD	Non-Excepted HOPD	Physician-Based and Freestanding
340B Drug Reimbursement	ASP − 22.5% [22]	ASP − 22.5%	-
Non-340B Drug Reimbursement	ASP + 6% [10,11]	ASP + 6%	ASP + 6%
CPT Reimbursement	^1^ HOPPS	HOPPS − 60% [6]	Physician fee schedule (50% of HOPPS)

^1^ Hospital Outpatient Prospective Payment System.

**Table 5:** Advisory Board Organic Growth Projections for Infusions for Health System Region.

Patient Population	Baseline	Year 5 Growth from Baseline	Year 10 Growth from Baseline
Chemotherapy	-	2.8%	7%
Non-Chemotherapy	-	3.9%	7.8%

**Table 6:** Covered Entities’ Projected Infusion Volume Growth.

Infusion Center	Baseline	Year 5 Growth from Baseline	Year 10 Growth from Baseline
Infusion Center A	-	11.8%	21.8%
Infusion Center C	-	3.8%	5.5%
Infusion Center D	-	13.4%	21.2%
Infusion Center B	-	8.9%	14.0%
Infusion Center E	-	10.1%	18.0%

**Table 7:** Chairs Available.

Chairs Available		
**Infusion Center**	**Weekday**	**Weekend**
Infusion Center A	17	
Infusion Center C	41	
Infusion Center D	8	8
Infusion Center B	5	
Infusion Center E	23	18

**Table 8:** Hours Open Daily.

Hours Open Daily		
**Infusion Center**	**Weekday**	**Weekend**
Infusion Center A	9	
Infusion Center C	9.8	
Infusion Center D	12	6
Infusion Center B	9	
Infusion Center E	12.5	8.5

**Table 9:** Annualized Appointments.

Annualized Appointments		
**Infusion Center**	**Weekday**	**Weekend**
Infusion Center A	9246	
Infusion Center C	17,260	
Infusion Center D	8428	920
Infusion Center B	4082	
Infusion Center E	17,566	2028

**Table 10:** Appointments Per Day.

Appointments Per Day		
**Infusion Center**	**Weekday**	**Weekend**
Infusion Center A	35.3	
Infusion Center C	65.9	
Infusion Center D	32.2	8.9
Infusion Center B	15.6	
Infusion Center E	67.0	19.7

**Table 11:** Appointments Per Chair Per Day.

Appointments Per Chair Per Day		
**Infusion Center**	**Weekday**	**Weekend**
Infusion Center A	2.1	
Infusion Center C	1.6	
Infusion Center D	4.0	1.1
Infusion Center B	3.1	
Infusion Center E	5.0	1.1

**Table 12:** Average RN FTE Per Day.

Average RN FTE Per Day		
**Infusion Center**	**Weekday**	**Weekend**
Infusion Center A	6	0
Infusion Center C	14	0
Infusion Center D	5.5	1.5
Infusion Center B	2	0
Infusion Center E	9.5	2

**Table 13:** Appointments Per RN FTE Per Day.

Appointments Per RN FTE Per Day		
**Infusion Center**	**Weekday**	**Weekend**
Infusion Center A	5.9	
Infusion Center C	4.7	
Infusion Center D	5.8	6.0
Infusion Center B	7.8	
Infusion Center E	7.1	9.8

**Table 14:** Infusion Centers’ Projected Growth in Appointments.

Infusion Centers’ Projected Growth in Appointments			
**Infusion Center**	**Baseline**	**Year 5**	**Year 10**
Infusion Center A	9246	10,337	11,262
Infusion Center C	17,260	17,916	18,209
Infusion Center D	9348	10,601	11,330
Infusion Center B	4082	4445	4653
Infusion Center E	19,594	21,573	23,121

**Table 15:** Preferred Strategy for Site of Care.

Preferred Strategy for Site of Care			
340B Classification	DSH	RRC	Neutral
Provider Groups	Heme/Onc/BMT	Pulmonology	Infectious Disease
	Gastroenterology	Dermatology	Pain
	Neurology	Allergy	
	Rheumatology	Nephrology	

**Table 16:** Payer Mix by Infusion Center.

Payer Mix by Infusion Center					
	Infusion Center A	Infusion Center B	Infusion Center C	Infusion Center D	Infusion Center E
Commercial	43.8%	17.7%	24.4%	25.5%	19.7%
Health System Commercial	21.6%	19.7%	15.8%	18.5%	24.6%
Medicaid	3.6%	22.4%	6.6%	8.2%	9.0%
Medicare	28.7%	37.4%	50.3%	44.5%	46.1%
Self-Pay	0.5%	0.7%	1.6%	1.4%	0.5%
Other	1.8%	2.0%	1.3%	1.9%	0.1%

**Table 17:** Projected Chairs Needed.

Projected Chairs Needed			
Metric	Baseline	Year 5	Year 10
Appointments	59,590	64,940	68,648
Number of Chairs	94	101	107

**Table 18:** Projected Chair Type Needed.

Projected Chair Type Needed			
Chair Type	Baseline	Year 5	Year 10
HOPD	77	82	86
Non-HOPD	17	19	21
Total	94	101	107

**Table 19:** Projected Chairs Needed by Provider Group.

Projected Chairs Needed by Provider Group						
	Baseline		Year 5		Year 10	
Provider Group	Appointments	Chairs	Appointments	Chairs	Appointments	Chairs
Allergy	498	0.6	549	0.6	589	0.7
Cardiology	36	0.0	41	0.0	44	0.0
Dermatology	170	0.2	187	0.2	201	0.2
Endocrinology	668	0.8	736	0.9	789	0.9
Family Medicine	1076	1.3	1187	1.4	1273	1.5
Gastroenterology	3558	4.5	3928	4.9	4218	5.3
Hematology/Oncology	30,148	59.4	32,401	63.1	33,862	65.6
Infectious Disease	5294	6.1	5845	6.7	6263	7.2
Internal Medicine	1462	1.8	1613	2.0	1731	2.1
Nephrology	1518	2.3	1694	2.6	1832	2.8
Neurology	2922	3.5	3220	3.9	3453	4.2
Other	602	0.7	664	0.8	711	0.8
Pain	7274	7.7	8065	8.6	8522	9.0
Pulmonology	444	0.5	493	0.6	529	0.6
Rheumatology	3920	4.7	4318	5.1	4630	5.5
Total	59,590	94.0	64,940	101	68,648	107

**Table 20:** Original Infusion Center F Planning.

Original Infusion Center F Planning		
**Infusion Center**	**HOPD Chairs**	**Non-HOPD Chairs**
Infusion Center C	0–41	
Infusion Center D	8	
Infusion Center B	5	
Infusion Center E	23	
Infusion Center F	72	
Home Infusion Provider		6
Total	108–149	6

**Table 21:** Updated Infusion Center F Planning.

Updated Infusion Center F Planning		
**Infusion Center**	**HOPD Chairs**	**Non-HOPD Chairs**
Infusion Center C	0–41	
Infusion Center D	8	
Infusion Center B	5	
Infusion Center E	23	
Infusion Center F	50	22
Home Infusion Provider		6
Total	86–127	28

**Table 22:** Projected Capacity of New Infusion Centers.

Projected Capacity of New Infusion Centers		
Metrics	Infusion Center F	Home Infusion Provider
Number of Chairs	72	6
Appointments Per Year	31,786	4741
Appointments Per Day	121.3	18.1
Appointments Per Chair Per Day	1.7	3.0

**Table 23:** Planned Home Infusion Provider Lidocaine Infusions’ Ramp Up.

Planned Home Infusion Provider Lidocaine Infusions’ Ramp Up		
Month	Patients	Infusions
0	3	9
1	8	24
2	13	39
3	18	54
4	23	69
5	28	84
6	33	99

## Data Availability

The data are not publicly available as they contain PHI and proprietary information.
